# Garlic (*Allium sativum*)-derived SEVs inhibit cancer cell proliferation and induce caspase mediated apoptosis

**DOI:** 10.1038/s41598-021-93876-4

**Published:** 2021-07-20

**Authors:** İrem Özkan, Polen Koçak, Merve Yıldırım, Naz Ünsal, Hazal Yılmaz, Dilek Telci, Fikrettin Şahin

**Affiliations:** Department of Genetics and Bioengineering, Faculty of Engineering and Architecture, Yeditepe University, 26 Ağustos Campus, Kayisdagi Cad., Kayisdagi, 34755 Istanbul, Turkey

**Keywords:** Molecular biology, Molecular medicine

## Abstract

As a key component of the cell-to-cell communication, small extracellular vesicles (SEVs) released from various sources are known to be affecting the physiological conditions of the target cells. Although it has been suggested that edible plant-derived nanoparticles contributes to the cross kingdom communication with the mammalian cells, the effect of these particles on cancer cell progression still needs a further exploration. Here, we isolated and then characterized garlic derived SEVs by nanoparticle tracking analysis, electron microscopy and SEV surface antibodies. In order to investigate anti-cancer property of garlic SEVs A498 human kidney carcinoma, A549 human lung carcinoma were used as cell models along with the normal human dermal fibroblast cell lines. Annexin V/pI staining and analysis of apoptotic mRNA and protein expression levels suggested that garlic SEVs induced apoptosis through activation of intrinsic pathway. Furthermore, angiogenic VEGF protein expression levels significantly decreased in response to SEVs treatment in cancer cells. Our results support that garlic derived SEVs could cause apoptotic cell death among cancer cells while normal cells remain unaffected with the treatment. This study revealed for the first time that plant SEVs possess anti-cancer affects by inducing caspase mediated apoptosis and provided a new alternative for cancer treatment.

## Introduction

In order to maintain tissue homeostasis as well as the integrity of the organisms, cell to cell communication serves a significant purpose^1^. Extracellular vesicle mediated communication between cells has recently become a dominant area of research among scientists due to their unique ability to shuttle variety of different molecules from producing cells to target cells^2^. Previously thought as garbage bins of the cells, exosome like nanoparticles are lipid complexes composed of multiple 3′–5′ exonucleases that work together in a harmony through a functional unit^3,4^. They are now comprehended as highly regulated and organized structures with a size range between 30 and 150 nm^5^. Even though proteins, lipids, DNA fragments, mRNA and surface markers are all common cargo for SEVs, the specificity of these molecules vary based on the cell type they are secreted from^6^. Accumulating evidence suggest that exosome like nanoparticles have role in the RNA processing as well as RNA turnover. The ability to destroy malfunctioning materials without harming the functional RNA indicate that extracellular vesicles and their microRNA composition could be used as a diagnostic tool and a therapeutic tool inside the cell^7^.

Recent studies have suggested that plants do also secrete extracellular extracellular vesicles involved in the cellular communication of plants^8^. These plant derived extracellular vesicles have similarities with mammalian derived extracellular vesicles with respect to their morphology as well as their release mechanism and content^9^. More particularly, exosome like extracellular vesicles secretion is known to be mediated by multivesicular body secretion outside of the cell due to the fact that their number increase in the presence of stress factor^10^. According to the literature, macrovesicles play a role in defence mechanism of the barley leaves when they face powdery mildew fungus attack^11^. Furthermore, exosome like extracellular vesicles derived from edible plants (grape, grapefruit, ginger, carrot) exert anti-inflammatory effect during the inflammatory bowel disease^12^. Molecular composition of eukaryotic exosome like extracellular vesicles and plant derived extracellular vesicles are not entirely the same in the structure, but certain proteins like aquaporins, and surface proteins such as heat shock proteins (HSP70), CD63, CD81 along with phosphatidic acids (PA) enriched lipids are common between these two structures^13^. On the other hand, miRNA levels as well as lipid compositions differ between mammalian and plant derived extracellular vesicles. While cholesterol levels are higher in mammalian derived extracellular vesicles, their phospholipid levels are less than plant derived extracellular vesicles which are composed of ~ 98% phospholipids^14^. Although the underlying mechanisms of how plant derived extracellular vesicles operate are still unknown, they are shown to have anti-proliferative and anticancer activities^15–18^.

Herein, we show that the juice of *Allium sativum* contains small extracellular vesicles, SEVs that have anti-proliferative and apoptotic activities on human kidney carcinoma cell line A498 and human lung carcinoma cell line A549 cell lines whereas no significant toxicity detected on the healthy human dermal fibroblast cells in vitro*.* All in all, a brand new approach for cancer treatment, developed from using natural, edible plant species, might be emphasized with our findings.

## Materials and methods

### Cell culture

Human kidney carcinoma cell line A498 (ATCC HTB-44) and human lung carcinoma cell line A549 (ATCC CCL-185) were obtained from American Type Culture Collection and was cultured in complete high glucose Dulbecco's Modified Eagle Medium (DMEM) whereas the primary human dermal fibroblast cells (HDF) were cultured in complete low glucose Dulbecco’s Modified Essential Medium (DMEM), supplemented with 10% (v/v) FBS (Fatal Bovine Serum) and 100 units/ml Penicillin/Streptomycin/Amphotericin (1%) antibiotics (Invitrogen, Gibco, UK). Cells were grown on T25, T75, T125 flasks which were incubated in Nuaire NU5510/E/G incubator at 37 °C in a highly-humidified atmosphere (RH 80%) with 5% CO_2_ rate and 95% (v/v) air.

### Garlic derived SEVs preparation and identification

SEVs-like vesicles were isolated from *Allium sativum*. Garlic was purchased from Taşköprü, Kastamonu, Turkey. Garlic was minced and grounded in a laboratory blender to obtain the juice, which was then centrifuged at 300 g for 10 min. Following transfer of the supernatant into new tubes and SEVs isolation by using aqueous two-phase systems is performed as described^19^. In brief, 20 ml of garlic juice is mixed with ATPS-EV isolation solution in 1:1 ratio and centrifuged at 1000×*g* for 10 min. After the centrifuge, 80% of the solution is discarded and PEG rich washing solution is added and centrifuged at 1000×*g* for 10 min and then repeated one more time. Following the isolation, SEVs were filtered with 0.22 μm pore filter to be used during the experiments. Quantification of the SEVs like vesicles was determined with the Lowry Assay (Bio Rad, USA). Identification of the isolated SEVs were done by scanning electron microscopy (SEM), dynamic light scattering and flow cytometry. For investigation using scanning electron microscopy, samples swapped on a glass microscope slide were dried at − 20 °C overnight and then coated (Belter SCD 005 Sputter Coater, Australia) prior to microscopic examination (Carl ZEISS EVO 40, Germany). For flow cytometry analysis, isolated SEVs were labelled with anti-CD9, anti-CD63 and anti-HSP 70 antibody (Abcam, UK) and analyzed using Becton Dickinson FACS Calibur flow cytometer.

### SEVs characterization via nanoparticle tracking analysis and zetasizer

Size distribution and particle number of the isolated SEVs was determined by Nanosight and zetasizer (Malvern Instruments Ltd, Malvern, Worcestershire, UK). SEVs samples were diluted in 1 ml distilled water and parameters were analyzed at room temperature. All samples were evaluated in at least 4 replicates. Blue laser beam at 488 nm was applied to the SEVs solution and a video was recorded with frame rate of 25 frames/s. The video was analyzed by NTA software version 3.3, Nanosight. Each SEVs replicate was analyzed at least 5 times, and their Brownian motion as well as their particle movement velocity were used to analyze the particle size of SEVs by using two-dimensional Stokes–Einstein equation. Zetasizer Nano ZS (Malvern Instruments) was also used to evaluate particle size of the isolated SEVs.

### Cell viability assay

Cells were seeded with a concentration of of 5 × 10^3^ cells/well in a 96 well plate and treated with escalating doses of *A. sativum* nanovesicles and garlic juice (5–50 μg/ml) for 24, 48 or 72 h in a humidified incubator (5% CO_2_ in air at 37 °C). The cell viability was performed with WST-1 assay (BioVision, CA) as described previously^20^. The absorbance was evaluated with the spectrophotometer at 540 nm. Three independent experiments were performed to generate the percentage of growth versus untreated control cells.

### Annexin V assay

The apoptotic effect of garlic SEVs on cancer cells was detected with Annexin V Detection Kit (BioLegend, USA) according to the manufacturers’ protocol. A498, A549 and HDF cells were seeded onto 6-well plates with the cell density of 1 × 10^5^ and treated with 50 μg/ml of *A. Sativum* SEVs for 24 and 48 h. Following the incubation process, cells harvested were incubated with Annexin V FITC and PI PE conjugated antibody and Apoptosis and necrosis were evaluated using flow cytometer. Obtained data was then analyzed using CellQuest Pro program.

### RNA extraction and real-time PCR

Total RNA Purification Plus Kit (Norgen, Ca) was used to perform total RNA isolation from cells treated with garlic SEVs according to the manufacturer’s protocol. QuantiTect Reverse Transcription Kit (Qiagen, France) was used for the conversion of isolated RNA into cDNA. QuantiTect SYBR Green PCR kit (Qiagen, France) was used to analyze the mRNA expression level of Bcl-2, Bax, P53, Cas 3 and Cas 9 genes (Table 1). Reaction mixture consisted of SYBR Green PCR mix, universal primer, RNase-DNase free water and 500 ng for each sample, and reactions were conducted according to the manufacturer’s protocol with iCycler RT-PCR system (Bio-Rad, Hercules, CA). During the analysis 18S rRNA reference gene was used to make relative quantification. The standard curve was used to analyze absolute quantification.

**Table 1:** Sequences of primers used in RT-PCR assays.

Marker	Sequences
18S	F 5′GTAACCCGTTGAACCCCATT3′ R 5′CCATCCAATCGGTAGTAGCG3′
P53	F 5′TTACTCCCTCCATCTCCACC3′ R 5′TCATCAAACCCTTCAGCCAG3'
Caspase-9	F 5′GTGGACATTGGTTCTGGAGGAT3′ R 5′ GTGGACATTGGTTCTGGAGGAT3′
Caspase-3	F 5′GAGGCGGTTGTAGAAGAGTTCGTG3′ R 5′TGGGGGAAGAGGCAGGTGCA3′
Bax	F 5′TGGAGCTGCAGAGGATGATTG3′ R 5′GAAGTTGCCGTCAGAAAACATG3′
Bcl-2	F 5′CCTGTGCACCAAGGTGCCGGAACT3′ R 5′CCACCCTGGTCTTGGATCCAGCC3′

*Bax* Bcl-2 associated X protein.

### Cell cycle analysis

HDF, A498 and A549 cells were seeded with the concentration of 1.5 × 10^5^ cells/well onto 6-well plates and incubated for 24 and 48 h at cell culture conditions. In order to synchronize cell cycle, cells were treated with serum free medium for 24 h followed by treatment of 200 µg/ml garlic SEVs. Cells were then detached with trypsin and centrifuged cells were washed with PBS solution. Cells were fixed with 70% EtOH at − 20 °C for 2 h and treated in PBS containing 1% Nonidet (AppliChem, Spain) and 0.1% RNase A (Invitrogen, UK) at 37 °C for 30 min. Finally, cells were incubated with 1 µg/ml Propidium Iodide at the dark room and cell cycle distribution was then analyzed by flow cytometer.

### Enzyme-linked immunosorbent assay

Cells seeded at 2 × 10^5^ cells/well into 6-well plates were treated with 50 μg/ml SEVs for 48 h. Following cell lysis in RIPA buffer containing 1% protease inhibitor cocktail for 5 min on ice, lysates were centrifuged at 14 000×*g* for 15 min at 4 °C. The concentration of proteins were measured with Lowry Assay as described in the manufacturer’s instructions. (Bio-Rad USA). 50 µg protein from each sample (in 100 µl) is transferred to the precoated ELISA plates and incubated at + 4 °C overnight. The total amount of cleaved caspase 3 (Ray Biotech, USA) and Bcl-2 (Thermo Fisher Scientific, USA) protein expressions were quantified and normalized as micrograms of Cas3 or Bcl-2 per millilitre of total protein content using ELISA kits as described previously in the manufacturer’s guide. For VEGF Elisa**,** medium of control cells and treated cells were collected and centrifuged at 10,000 rpm for 30 s in order to remove any impurities remained in the media and then the amount of VEGF secretion was quantified and normalized as micrograms per milligrams of total protein content using ELISA kit according to manufacturer’s guide (Thermo Fisher Scientific, USA).

### Western blotting

The caspase 3 protein expression in garlic SEV-treated cells was confirmed using the Wes Simple Western apparatus (Protein Simple, San Jose, USA). Denatured 0.4–0.6 g protein was loaded onto Wes multiwell plates after being denatured in a DTT-based buffer supplied by the manufacturer. Protein isolation, antibody binding, and labeling were all accomplished using capillary cartridge separation.

### Cellular reactive oxygen species detection

Intracellular ROS levels were measured by the DCFDA/H2DCFDA—Cellular ROS Assay Kit (Abcam, USA). A549 and A498 cells (8 × 10^3^ cells/well) were seeded in black 96-well plate and incubated at 37 °C overnight. 24 h later cells were either given 15, 20, 25, 30, 35, 40 and 50 μg/ml garlic SEVs, control cells were treated with only growth medium. Following day, media was removed and 100 μl 1× buffer was placed into each well and cells were stained by DCFDA solution for 45 min at 37 °C in the dark. After removal of DCFDA solution, 1× buffer was added on each well. Fluorescent intensity was measured immediately on Multiscan spectrophotometer (Biotek, USA) at Ex/Em = 485/535 nm.

### Statistical analysis

One-way analysis of variance (ANOVA) on percentage and Graph-Pad Prism 6 (Graph-Pad, La Jolla, CA) software were used for the statistical analysis. The values of *p* ≤ 0.05 were considered statistically significant.

## Results

### Characterization of plant SEVs-like nano vesicles

**Figure 1:**
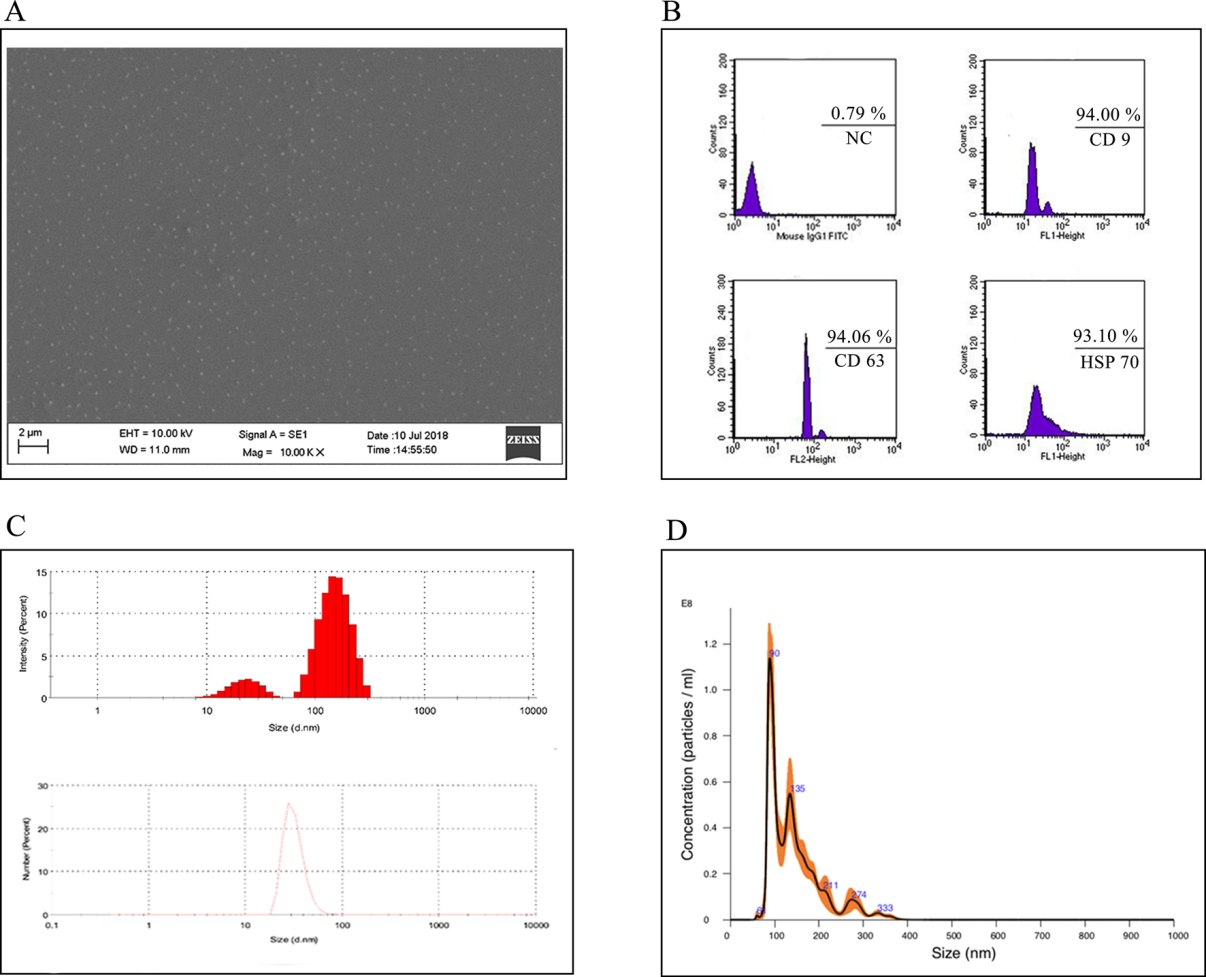
Characterization of garlic exosomes isolated with ATPS method. Scanning electron microscope (**A**) image shows irregularly formed various nano vesicles with the scale bar indicating 2 µm. Characterization of garlic SEVs (**B**) via flow cytometry analysis with the extracellular vesicle cell surface markers against CD9, CD63, and HSP 70 antibodies. The size distribution of garlic exosomes (**C**) via dynamic light scattering analysis and (**D**) nanoparticle tracking analysis (NTA) demonstrate the size of the nanoparticles ranging from 50 to 150 nm.

Garlic SEVs were isolated from the garlic juice using aqueous two phase system, ATPS method as described. Scanning electron microscope analysis indicated the size of the isolated SEVs to be < 150 nm (Fig. 1A). SEVs characterization was also confirmed by flow cytometry. Garlic derived nanovesicles expressed 93.10% HSP 70, 94.00% CD9, 94.06% CD63 SEVs specific markers as shown in Fig. 1B. Zetasizer (Fig. 1C) and Nano sight (Fig. 1D) analysis displayed 50–150 nm diameter for SEVs. Taken together, our data showed that nanovesicles identified in garlic juice were SEVs-like based on their size and CD marker profiles.

### Effect of garlic SEVs on the cell survival of A498, A549 and HDF cells

**Figure 2:**
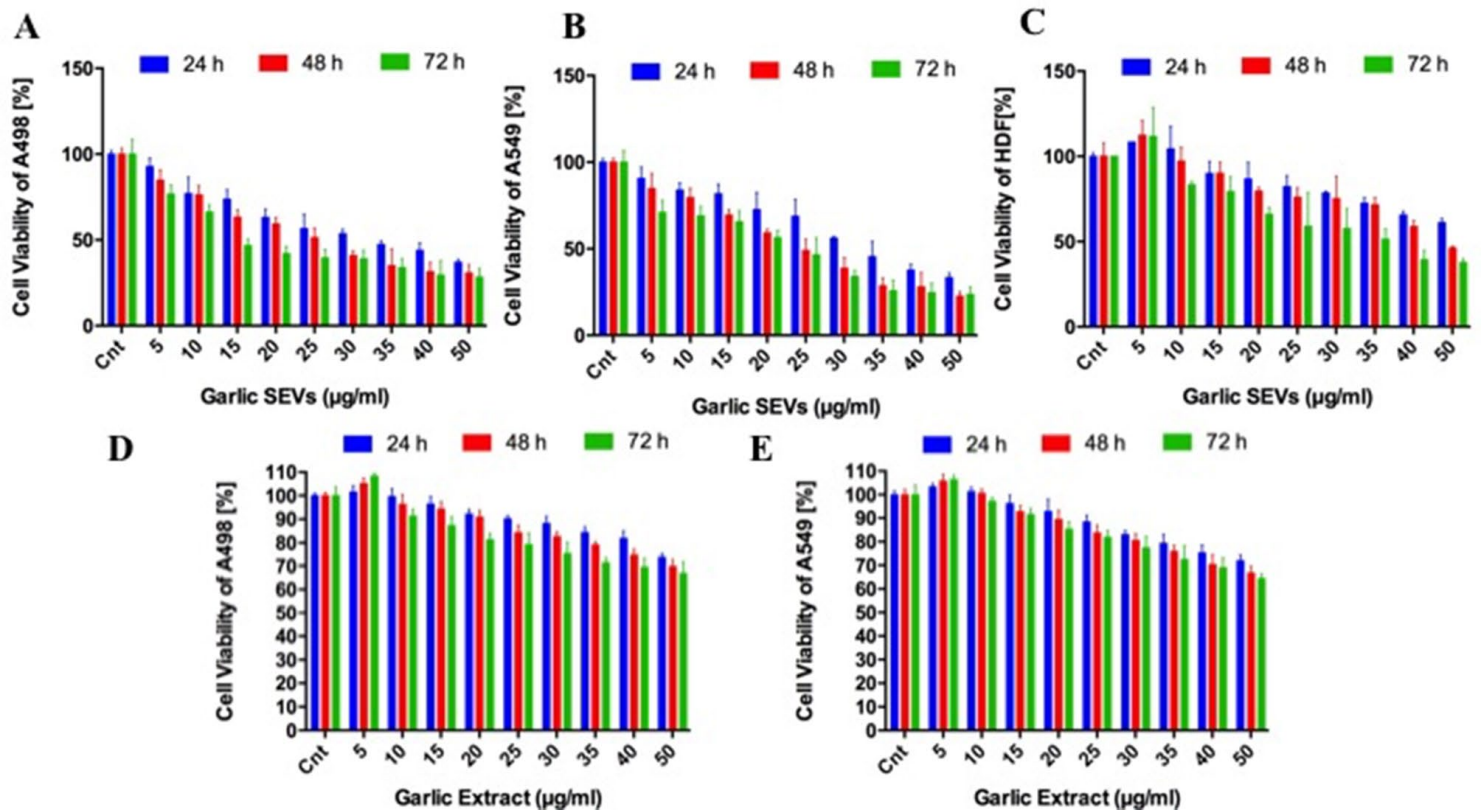
Analysis of the cell viability for human kidney carcinoma, A-498 (**A**), human lung carcinoma, A-549 (**B**) and normal human dermal fibroblast (HDF) cell line (**C**) when treated with varying concentrations (5–50 µg/ml) of garlic exosome and analysis of the cell viability for human kidney carcinoma, A-498 (D) and human lung carcinoma, A-549 when treated with varying concentrations (5–50 µg/ml) of garlic juice (**E**). Cells were seeded on 96-well plates and allowed to attach overnight. MTS assay was performed at 24, 48, and 72 h incubation (37 °C, 5% CO_2_) in DMEM supplemented with 10% FBS. Cell death was analyzed by correlating the absorbance value of nontreated control cells to 100. The percentage of cell viability was calculated by assigning the absorbance value obtained from NC: negative control, standard growth medium treated cells as 100%.

The effect of various concentrations (5–50 μg/ml) of garlic SEVs on A498, A549 and HDF cell viability were determined at 24, 48 and 72 h (Fig. 2). Concentrations up to 50 μg/ml of garlic SEVs exerted significant cytotoxic effects on both cancer cell lines where the viability of A498 and A549 cells decreased down to 35% within 24 h. The cytotoxic effect of garlic SEVs increased in the following 48 h and 72 h by 28% and 22%, respectively (Fig. 2A,B). Interestingly, increasing doses of the garlic SEVs showed no significant cytotoxic effect on the viability of normal HDF cells. Contrarily, the proliferation of HDF cells was significantly increased up to 188% when exposed to garlic SEVs at low doses (Fig. 2C). Moreover, to check the effect of garlic juice on these cancer cells, garlic juice was used in treatment of these cells on similar doses.

### Treatment of garlic SEVs caused S phase cell cycle arrest in A498 and A549 cells

**Figure 3:**
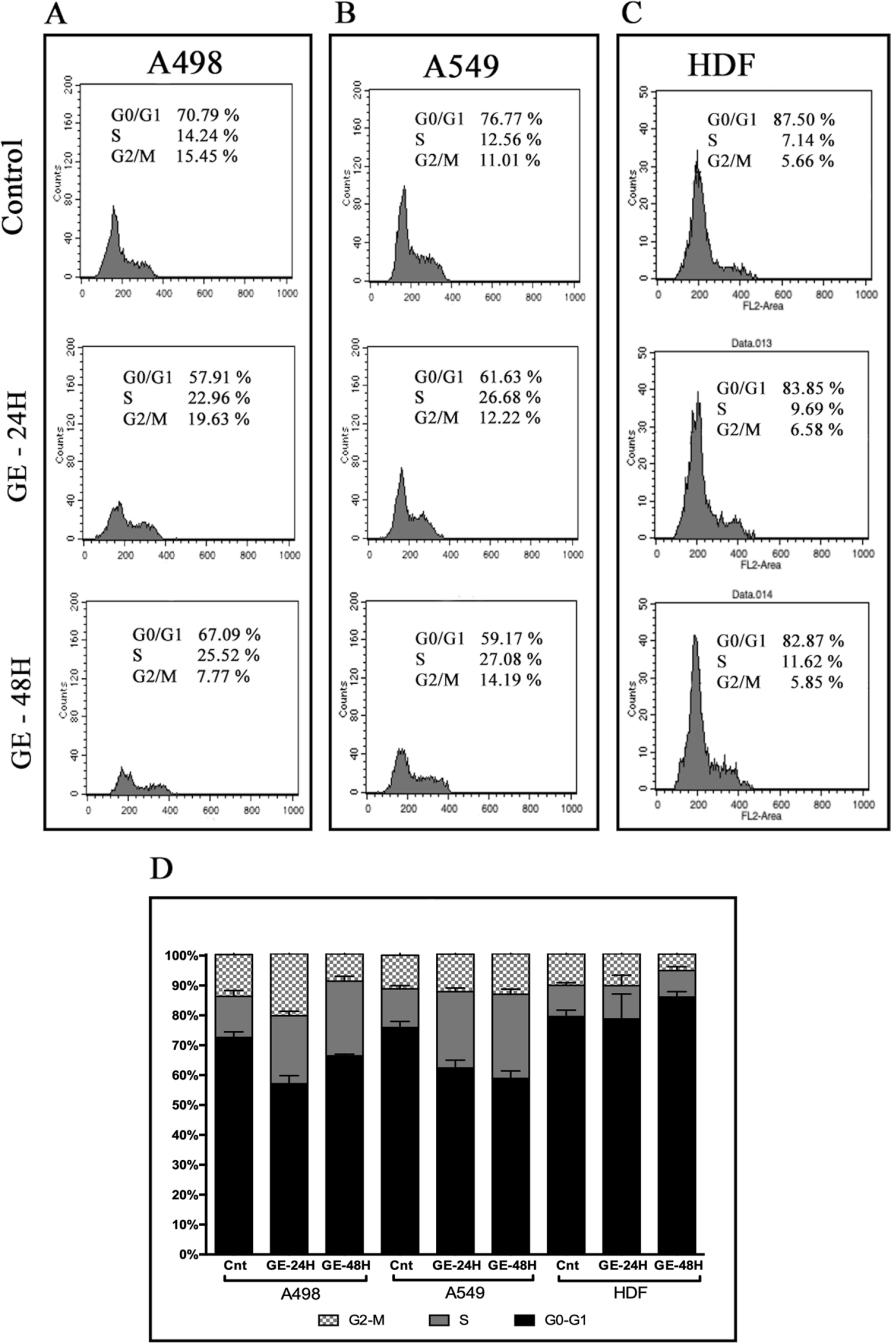
Cell cycle distributions of human kidney carcinoma A-498, human lung carcinoma A-549 and human dermal fibroblast HDF cells (**A**) and their representative bar graph (**B**) after treatments with garlic SEVs for 24 and 48 h. Cells were seeded on 6-well plates and allowed to attach overnight. Cells were treated with 50 µg/ml garlic exosome and incubated for 24 and 48 h and then they were subjected to cell cycle analysis as described in the “Materials and methods” section 50 µg/ml garlic SEVs treated cell populations at G0/G1, S and G2/M phases were analyzed. Data expressed as the mean ± SD.

The effect of garlic SEVs on cell cycle progression was also investigated on A498, A549 and HDF cells via flow cytometry in order to clarify the mechanism contributing the growth inhibition. 24 and 48 h treatment with garlic SEVs led to accumulation of both cancer cells in S phase (Fig. 3). The number of A498 cells in G0/G1 phase significantly decreased from 70 to 57% in the first 24 h. Consistently, 24 h after the treatment with garlic SEVs, A498 cells displayed an increase in the S phase from 14 to 22%. Although the treatment led to a slight increase in the G2/M phase (from 15 to 19%), at 48 h S phase accumulation reached to 25% whereas the accumulation in the G2/M phase drop down to 7% among A498 cells (Fig. 3A). Similarly, a respective decrease of 15% and 17% in the number of G0/G1 phase A549 cells were observed when treated with garlic SEVs for 24 and 48 h. An increase in S phase cell population from 12 to 26% for 24 h and to 27% for 48 h was recorded for A549 cells in the presence of garlic SEVs. Change in G2/M phase A549 cells was recorded as 1% for the 24 and 48 h treatments. In contrast to cancer cells, only a slight increase of 2% in the S phase cell population for HDF cells was recorded 24 and 48 h after the treatment with garlic SEVs. These results showed that garlic SEVs led to a cell cycle blockage on both A498 and A549 cells compared to their respective non-treated control. On the contrary, garlic SEVs did not lead to a significant change in the cell cycle rate of dermal fibroblast cells.

### Apoptotic effect of garlic SEVs on A498, A549 and HDF cells for 24 and 48 h

**Figure 4:**
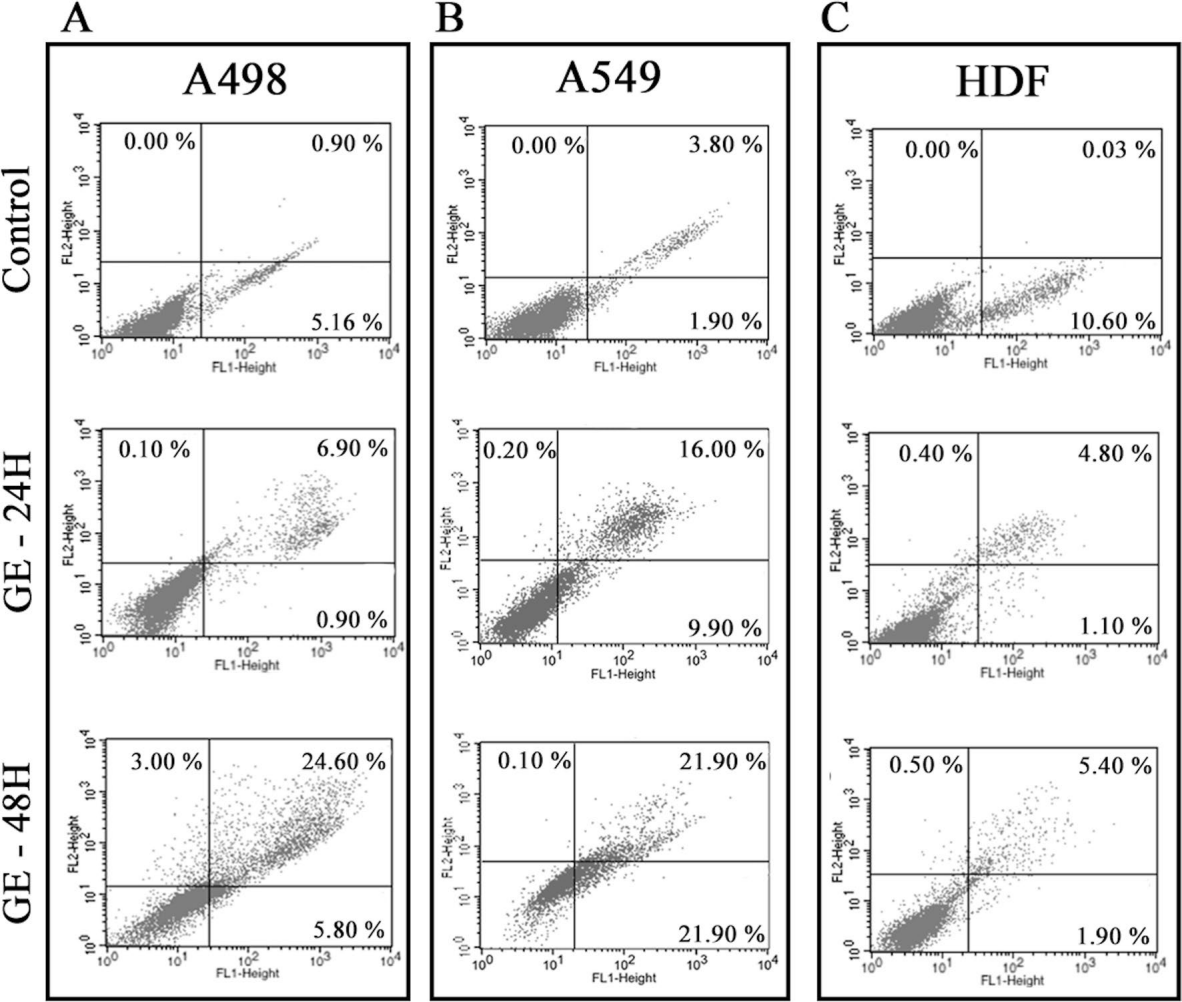
Annexin V/pI costaining assay was performed on human kidney carcinoma A-498, human lung carcinoma A-549 and human dermal fibroblast (HDF) cells in order to detect apoptosis after the treatment of 50 µg/ml garlic SEVs for 24 and 48 h. Early and late apoptosis quantifications were presented. Data expressed as the mean ± SD.

In order to further evaluate the cell cycle arrest and cell death mechanism, Annexin V-FITC/pI assays was performed to confirm the apoptotic effect of garlic SEVs on A498, A549 cells. 50 µg/ml of garlic SEVs treatment on A498 cells showed that apoptotic cell death was nearly 7% on 24 h which was increased to 24% after 48 h treatment. No significant necrosis was observed in neither 24 nor 48 h treatment (Fig. 4A). A higher rate of apoptosis with 16% was evident in A549 cells when treated with 50 μg/ml of garlic SEVs at 24 h. The apoptotic cell death ratio was increased to 21% in A549 cells at the end of 48 h due to SEVs treatment (Fig. 4B). On the other hand, no significant induction of apoptosis was observed for normal HDF cells with the apoptotic cell ratio remaining at 4% and 5% for the 24 and 48 h treatments in response to garlic SEVs (Fig. 4C). Increased ROS levels and mitochondrial dysfunction, which are seen in many cancers, cause oxidative damage to biological systems, particularly in genomic and mitochondrial DNA, resulting in oncogene activation and inactivation of tumor suppressor genes, which contributes to cellular transformation. ROS levels were evaluated by the DCFDA / H2DCFDA—Cellular ROS Assay. Results showed that garlic SEVs treatment resulted in an increase in ROS levels.

### Effect of garlic SEVs on pro-/anti-apoptotic genes and protein expression levels

**Figure 5:**
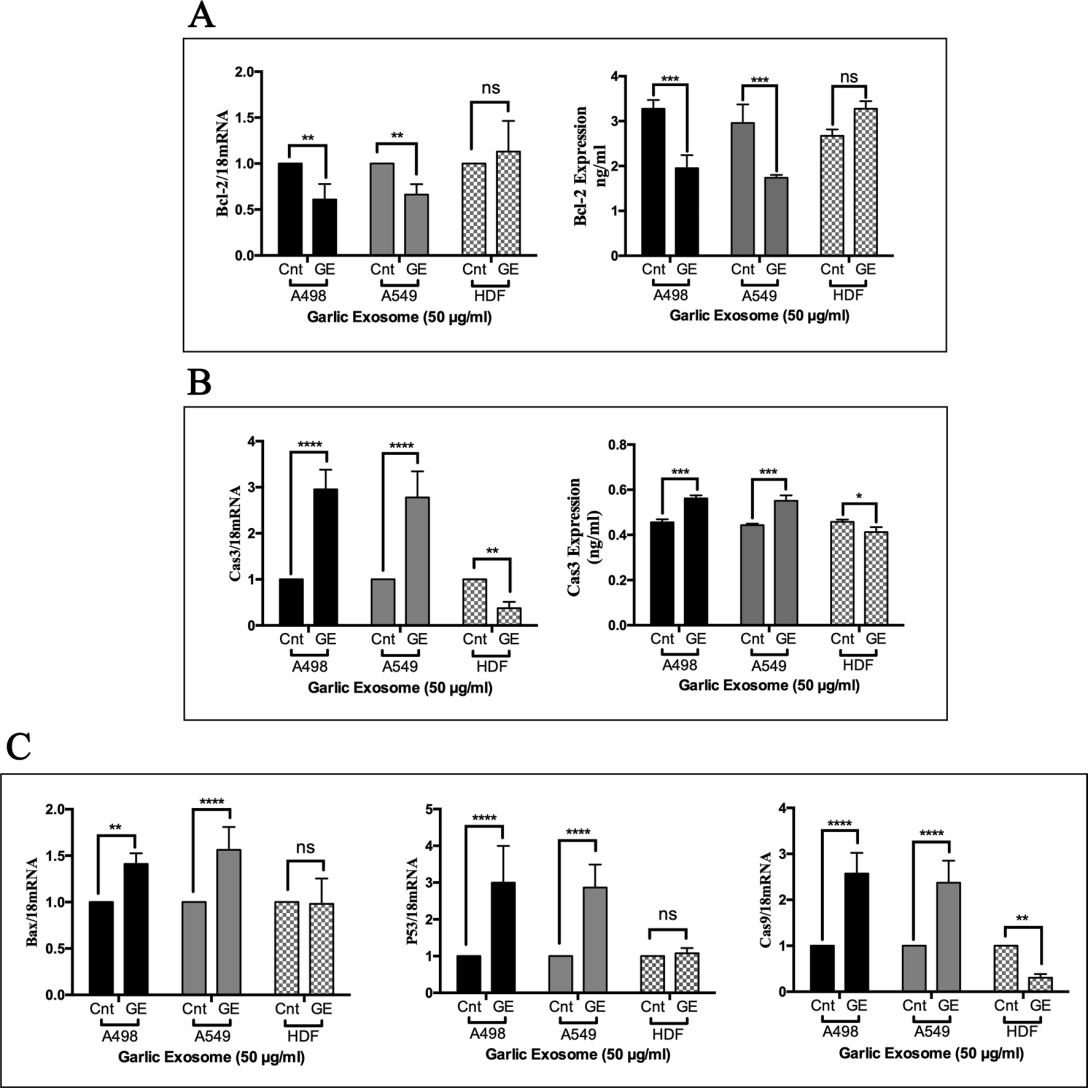
Quantitative RT-PCR analysis of Bcl-2, (**A**), Cas3 (**B**) Bax, P53, and Cas9 (**C**) expression levels of human kidney carcinoma A-498, human lung carcinoma A-549 and human dermal fibroblast (HDF) cells. Expression of 18S was used as a negative control and gene expression levels were normalized to the housekeeping gene 18S. Cells were plated in 6-wells and treated with 50 µg/ml garlic SEVs. After 48 h of treatment, RNA isolation and cDNA conversion is performed as described in the “Materials and methods” section. Data are expressed as the mean ± SD. Results are presented at n = 4 for each group one-way ANOVA multiple comparisons, **p < 0.01, ***p < 0.001, ****p > 0.0001, *ns* not significant.

Next, we examined the effect of 50 µg/ml garlic SEVs on pro- and anti-apoptotic gene expression levels using RT-PCR as well as their respective protein expressions levels by ELISA. Anti-apoptotic Bcl-2 gene expression level was significantly decreased down nearly to 60% in A498 cells and 65% in A549 cells. Although Bcl-2 gene expression level was increased up to 150% in normal HDF cells, this change was found to be statistically not significant. The change in Bcl-2 gene expression levels was confirmed with ELISA, showing that anti-apoptotic Bcl-2 protein levels were decreased nearly 40% in both A498 and A549 cells along with a significant increase up to 20% in normal HDF cells (Fig. 5A). Consistent with decreased anti-apoptotic gene and protein expression levels, there was a significant threefold increase in pro-apoptotic Cas3 gene expression levels in both cancer cells while sevenfold decrease was detected for Cas3 mRNA levels in normal HDF cells upon treatment. Caspase-3 protein expression levels have also risen up by 20% in cancer cell lines and reduced by 10% in HDF cells (Fig. 5B). In parallel to inclined Cas3 gene expression levels, a significant increase in pro-apoptotic genes such as Bax, P53 and Cas9 was also recorded for cancer cells. Both cancer cells exhibited a similar change in gene expression levels where a respective 1.5-, 3- and 2.5-fold increase in Bax, P53 and Cas9 genes was seen in response to the SEVs treatment. Albeit to these notable changes in cancer cells, normal HDF cells exerted no significant change in both Bax and P53 gene expression levels, while Cas9 gene expression levels was substantially decreased by sevenfold in HDF cells (Fig. 5C). Lastly, the Cas3 protein expression levels were evaluated by western blotting. Results demonstrated that garlic SEVs treatment lead to increase Cas 3 protein expression in A498 and A549 cells.

### Role of garlic SEVs on vasculogenesis

**Figure 6:**
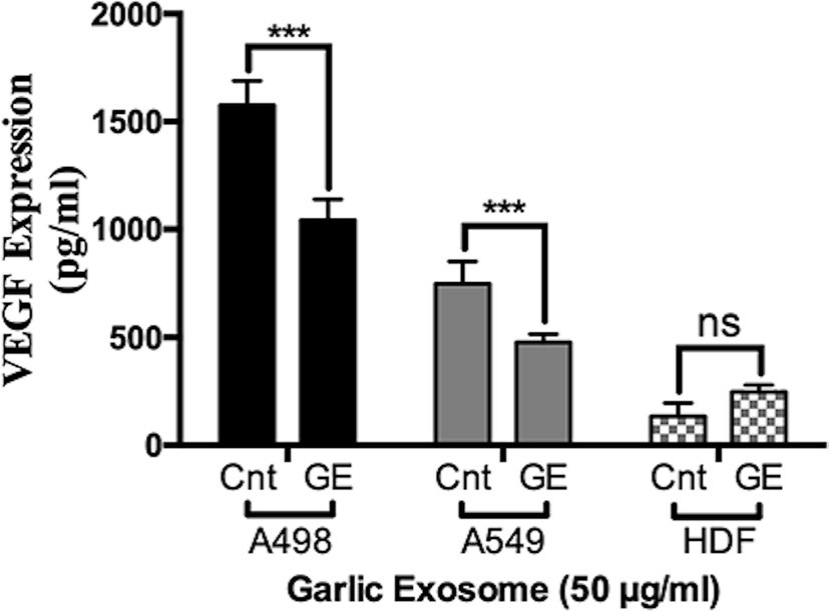
ELISA analysis of human kidney carcinoma A-498, human lung carcinoma A-549 and human dermal fibroblast HDF cells after the 48 h treatment of 50 µg/ml garlic SEVs. Cells were plated in 6-wells and treated with 50 µg/ml garlic exosome. Proteins of treated cells were harvested as described in the “Materials and methods” section. Data are expressed as the mean ± SD. Results are presented at n = 5 for each group one-way ANOVA multiple comparisons, **p < 0.01, ***p < 0.0001, *ns* not significant.

In order to determine the effect of garlic SEVs on vascularization, we evaluated the soluble VEGF protein expression levels on each cell line. The levels of VEGF in cell media was found the highest in A498 cells, followed by A549 and HDF cells (Fig. 6). VEGF secretion of A498 cells significantly decreased approximately to 1000 pg/ml levels from 1500 pg/ml. Similarly amount of VEGF released by A549 cells significantly decreased by 1.5-fold to 500 pg/ml levels in response to garlic SEVs. Although no significant change was observed in VEGF release after the treatment with garlic SEVs of HDF cell line, there was a slight increase up to 200 pg/ml in VEGF levels. (Fig. 6).

## Discussion

Comprehensive research for new therapeutic drugs for cancer have been an ongoing research topic during the last decades. Although several molecular compounds were studied distinctively both in vitro and in vivo for their chemotherapeutic and chemoprotective effects for various cancer types such as prostate, colon, breast and stomach carcinogenesis, the search for newer and more effective drugs is never-ending^21–24^. In this study, we characterized SEVs-like nanoparticles from garlic and evaluated their effect on both cancer and normal cells.

The use of natural extracts in cancer prevention and treatment has been an increasing trend as the numerous data in the traditional medicine suggest that these natural extracts possess anti-proliferative activities on different cancer types^25–27^. *A. sativum* had been used for external tumor treatment around 1550 B.C. by ancient Egyptians^28,29^. In order to determine whether garlic SEVs possess an anti-cancer effect, we chose 2 cancer cell lines A498 & A549 and one normal cell line, HDF, for evaluating the effects of garlic SEVs treatment on cell proliferation, cell cycle and cell survival.

Although plant EVs and SEVs are not as well studied as in mammalian cells, evidences supporting SEVs secretion from plants and their cross-kingdom effects are ever increasing. EV isolation and characterization from apoplastic fluid of Arabidopsis leaf and sunflower seeds among the top studies about exosomal characterization of plants^30,31^. Extensive size distribution (20–300 nm in size), protein, lipid and RNA contents from plants show similarities with mammalian SEVs. Recent literature suggested that the pre-observed effects of these natural extracts could be due to SEVs found in extract constituents^32^. Herein, we examined exosomal tetraspanin family marker proteins CD9, CD63 and HSP70 via flow cytometry from SEVs derived from garlic juice^8,12,31,33–36^. We also analyzed the size distribution and size intensity of the SEVs as well as scanning electron microscopy images of SEVs^37–39^.

Despite the lack of sufficient knowledge of interspecies communications of SEVs-like nanoparticles from plants, Chang et al. reported that anti-oxidant gene expression levels increased in intestinal macrophages after treatment with grape derived extracellular vesicles ^12^.

Hence, we evaluated if the SEVs derived from garlic possess any effect in a cross-kingdom communication and prevent cancer cell progression through consecutive in vitro studies such as MTS assay, cell cycle analysis, Annexin V/pI analysis, mRNA and protein expression levels. While the SEVs treatment significantly reduced the viability of cancer cells, it did not cause a cytotoxic response on the normal cell line HDF. One of the reasons that for this observed decrease in cell proliferation among cancer cells in response to SEVs treatment was the cell cycle arrest in the S phase with a non-significant changes in G2/M phase. Evidently, the treatment caused no significant cell cycle arrest on HDF which explains lack of anti-proliferative effect in response to the SEVs treatment.

In order to examine the relationship between cell cycle arrest and the mode of cell death mechanism, annexin-V/pI staining assay was performed for each cell line upon garlic SEVs treatment. treated cancer cells treated with garlic SEVs exhibited high apoptotic activities at all time points. Our results showed that, early and late apoptotic cell percentage has increased in a time dependent manner in both cancer cell lines whereas apoptotic cell number in normal cells remained not significant with respect to the control cells. Furthermore, pro- and anti-apoptotic pathways in each cell line were evaluated to demonstrate that these nanovesicles are in fact inhibiting cancer cell proliferation via certain pathways. Our results suggest that, treatment of kidney and lung carcinoma cells with garlic SEVs led to a significant increase in gene expression levels of pro-apoptotic genes such as p53, Bax, Cas3 and Cas9 while a statistically significant decrease in anti-apoptotic gene expression levels of Bcl-2 was detected in each cancer cell lines. ELISA results for Bcl-2 levels were found to be in line with the observed changes for Bcl-2 gene expression for A498 & A549 in response to SEVs treatment. Similarly, parallel results were obtained for Cas3 activation as increase in cleaved Cas3 levels was detected for SEVs treated A498 & A549. On the other hand, the change in the level of Bcl-2, p53 and Bax gene expression and Bcl-2 protein levels remained statistically not significant in HDFs upon treatment but a significant reduction in both pro-apoptotic Cas9 and Cas3gene expression as well as cleaved Cas3 was evident. Activated mainly by Cas9, Cas3 is one of the most vital executioner of the cellular apoptosis^40^, hence this finding suggest that the effect garlic SEVs was not limited to abrogated cancer cell proliferation but also to increased cell survival in normal cells. As a potent angiogenic factor, it is well established that the increase in VEGF expression contributes to the tumor angiogenesis in cancer cells as well as protecting these cells from apoptosis^41,42^. In response to treatment garlic SEVs, A498 & A549 displayed a significant decrease in VEGF secretion though no significant change was recorded for in normal human dermal fibroblast cells after the treatment with garlic SEVs.

In summary, data presented here strongly suggest that SEVs derived from garlic display therapeutic effects on cancer cells whilst they exert minimum to none cytotoxic effect to normal HDFs. On the contrary our results showed that these nanovesicles in fact increase the viability by increasing the anti-apoptotic Bcl-2 expression levels and decreasing the basal Cas3 activity. The ability of cross kingdom communication of these SEVs-like nanoparticles from garlic could be useful to develop new anticancer therapeutics with minimum side effects.

## Supplementary Information

Supplementary Figures.

## Abbreviations

*A. Sativum*: *Allium sativum*
HSP 70: Heat shock protein 70
miRNA: Micro RNA
VEGF: Vascular endothelial growth factor
PBS: Phosphate-buffered saline
