# Supplementary material for: Garlic (Allium sativum)-derived SEVs inhibit cancer cell proliferation and induce caspase mediated apoptosis
Source: Sci Rep. 2021 Jul 20;11:14773. doi: 10.1038/s41598-021-93876-4 (PMC8292337; doi:10.1038/s41598-021-93876-4)
Supplement: Supplementary file 1 — Supplementary Figures. [file 41598_2021_93876_MOESM1_ESM.docx]

**Supplementary Figures**


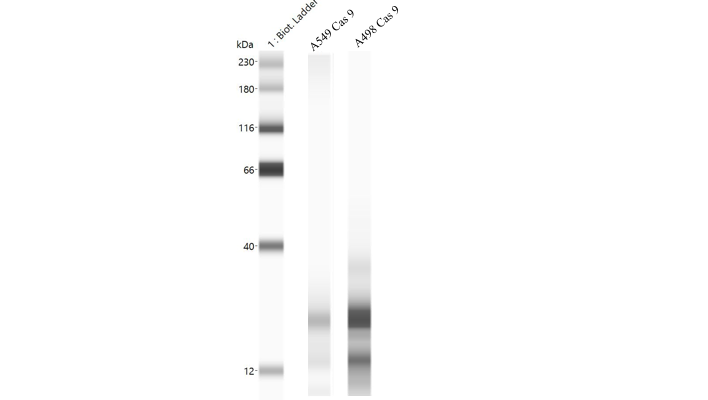


SFig1: Western Blot analysis of human kidney carcinoma A-498 and human lung carcinoma A-549 cells after the 48 hours treatment of 50 µg/ml garlic SEVs. Proteins of treated cells were harvested as described in the Materials and Methods section and Cas3 protein was evaluated with Simple Western ^TM^

^
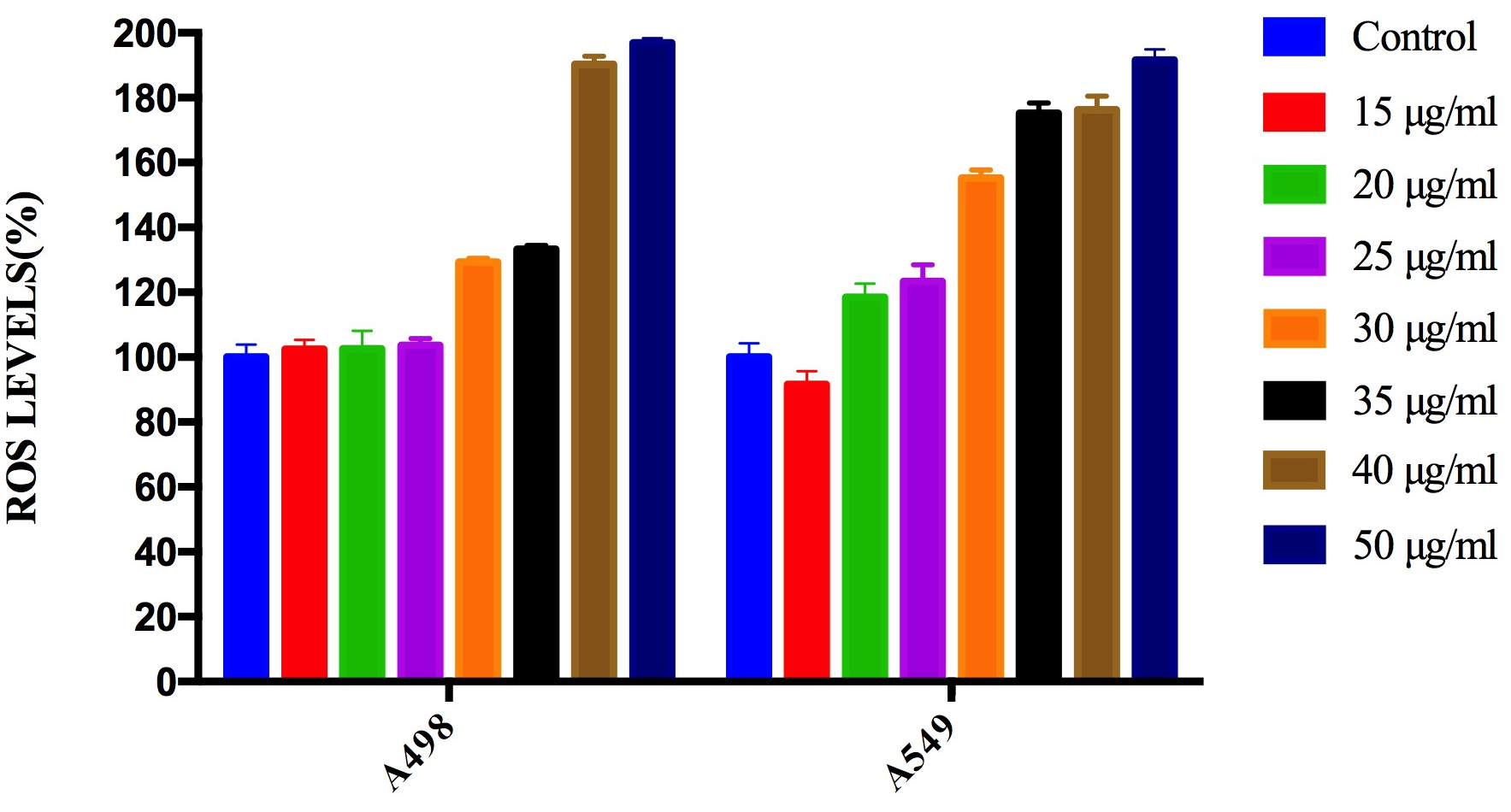
^

SFig2: ROS analysis of human kidney carcinoma A-498 and human lung carcinoma A-549 cells after the 48 hour treatment of 15-50 µg/ml garlic SEVs. ROS levels were evaluated by the DCFDA / H2DCFDA - Cellular ROS Assay.
